# Multi-Antigen Protein Vaccine Confers Protection in a Murine Model Against Intranasal *Haemophilus influenzae* Challenge

**DOI:** 10.3390/vaccines14040357

**Published:** 2026-04-17

**Authors:** Nouria Belkacem, Ala-Eddine Deghmane, Muhamed-Kheir Taha

**Affiliations:** Institut Pasteur, Université Paris Cité, Invasive Bacterial Infections Unit and National Reference Centre for Meningococci and Haemophilus Influenzae, CEDEX 15, 75724 Paris, France

**Keywords:** NTHi, vaccine, P5, P26, mucosal immunity, intranasal immunization, respiratory infection, bacterial clearance

## Abstract

**Background**: Non-typeable *Haemophilus influenzae* (NTHi) is a major cause of acute respiratory tract infections and chronic airway disease, despite its clinical importance, no licensed vaccine is available, largely due to the extensive genetic and antigenic diversity among circulating isolates. We previously identified conserved outer membrane proteins capable of inducing systemic protection against NTHi. **Methods:** In this study, we evaluated whether a multi-antigen protein vaccine composed of conserved NTHi antigens (P5 and P26) could protect against pulmonary infection. Transgenic mice expressing human transferrin and factor H were immunized via the intraperitoneal or intranasal route and challenged intranasally with a clinical NTHi isolate. Bacterial clearance, antigen-specific mucosal and systemic antibody responses, and recruitment of innate immune cells to the airways were assessed. **Results:** Both immunization routes significantly reduced bacterial loads compared with controls. Vaccination induced robust mucosal and systemic IgG and IgA responses and enhanced early recruitment of macrophages, monocytes, dendritic cells, and neutrophils to the airways. Intranasal immunization elicited strong mucosal antibody responses and was associated with improved local bacterial clearance. **Conclusions:** These findings demonstrate that multi-antigen vaccines targeting conserved NTHi proteins can elicit effective mucosal and systemic immunity and represent promising candidates for the prevention against NTHi respiratory infections.

## 1. Introduction

*Haemophilus influenzae* (Hi) is a Gram-negative bacterium that colonizes the human upper respiratory tract and is a major cause of mucosal infections, including otitis media, sinusitis, bronchitis, and exacerbations of chronic obstructive pulmonary disease (COPD) [1,2]. In young children, NTHi is one of the leading bacterial causes of acute otitis media worldwide, while in adults, it contributes significantly to chronic airway inflammation and disease progression in COPD and bronchiectasis [3,4]. Based on the antigenic properties of its polysaccharide capsule, *H. influenzae* strains are classified into six encapsulated serotypes (a–f) and non-encapsulated, or non-typeable, isolates (NTHi). Unlike encapsulated *H. influenzae* type b (Hib), for which effective conjugate vaccines are available, NTHi lacks a polysaccharide capsule and exhibits substantial genetic and antigenic heterogeneity, complicating vaccine development. Following the widespread introduction of Hib conjugate vaccines, NTHi has emerged as a dominant cause of both mucosal and invasive *H. influenzae* disease. Epidemiological surveillance studies conducted in Europe and North America indicate a relative and absolute increase in NTHi-associated infections over the past two decades, particularly among infants, elderly individuals, and immunocompromised patients [5]. In addition to acute infections, NTHi is capable of forming biofilms on respiratory mucosal surfaces, promoting persistent colonization, resistance to antibiotics, and evasion of host immune responses, including complement-mediated clearance [6,7]. Biofilm formation contributes to chronicity and recurrent infections, and significantly reduces antibiotic efficacy, thereby complicating clinical management [8,9]. The increasing burden of NTHi disease, combined with rising antimicrobial resistance, underscores the urgent need for effective preventive strategies. The current treatment relies largely on antibiotics; however, the emergence of β-lactam resistance and alterations in penicillin-binding proteins have been increasingly reported [10]. Given the lack of a polysaccharide capsule, protein-based vaccines targeting conserved surface-exposed antigens represent a rational approach for overcoming NTHi diversity [11]. Several candidate antigens, including outer membrane proteins, adhesins, and proteins involved in complement evasion, have been investigated; however, no licensed vaccine against NTHi is currently available [12,13,14].

In a previous study [15], we identified the conserved outer membrane proteins P5 and P26 as promising vaccine candidates and demonstrated that a multi-component formulation induced robust systemic protection against homologous and heterologous NTHi isolates [15]. These antigens are widely distributed among clinical isolates and are functionally involved in host–pathogen interactions, including adhesion to epithelial cells and resistance to complement-mediated killing [12,13]. However, because NTHi primarily causes respiratory tract infections, it is critical to evaluate whether these vaccine candidates can also confer protection at mucosal sites. Protection against systemic infection does not necessarily predict efficacy at the respiratory mucosa, where local IgA responses and early innate immune activation play pivotal roles in pathogen clearance [16,17].

In the present study, we investigated the potential protection of a multi-antigen P5–P26 vaccine against pulmonary NTHi infection using a transgenic mouse model permissive for *H. influenzae* growth. We compared intraperitoneal and intranasal immunization routes to assess their impact on bacterial clearance, mucosal antibody responses, and early innate immune activation in the airways. By specifically addressing mucosal immunity and local protection, this work complements our previous systemic infection model and provides a more comprehensive preclinical evaluation of this vaccine strategy. Furthermore, evaluating intranasal delivery allows exploration of a vaccination approach that may be particularly suited for preventing respiratory colonization and infection.

## 2. Materials and Methods

### 2.1. Ethical Statement

Animal work in this study was carried out at the Institut Pasteur in strict accordance with the European Union Directive 2010/63/EU (and its revision 86/609/EEC) on the protection of animals used for scientific purposes. The protocol was approved by the Institut Pasteur Review Board, which is part of the Regional Committee of Ethics of Animal Experiments of Paris Region (Permit Number 75-1554), and the approval of the Institut Pasteur Board (Dap210087/N°APAFIS #34085-2021112212171298).

### 2.2. H. influenzae and Growth Conditions

The NTHi isolate (LNP31258) was obtained from the National Reference Centre for meningococci, and *Haemophilus influenzae* (NRCMHi) from the Institut Pasteur, which performs an institutional role in surveillance [18].

Isolate was cultured in chocolate + PolyViteX agar plates (bioMerieux, Craponne, France) at 37 °C in the presence of 5% CO_2_.

For infection experiments, bacteria were grown overnight on chocolate agar plates, harvested in sterile phosphate-buffered saline (PBS), and adjusted spectrophotometrically to the desired concentration. The inoculum was confirmed by serial dilution and plating to determine colony-forming units (CFU).

### 2.3. Experimental Active Protection of Vaccine Candidates in Mice

Transgenic mice expressing human factor H and human transferrin were used and the recombinant proteins were prepared as previously described [15]. These transgenic mice provide a physiologically relevant model for *H. influenzae* infection, as the bacterium specifically interacts with human transferrin for iron acquisition and human factor H to evade complement-mediated killing.

Female mice aged 6–8 weeks were used in all experiments. Animals were randomly assigned to experimental groups and housed under specific pathogen-free conditions with ad libitum access to food and water.

Active protection was evaluated by immunizing mice with purified recombinant proteins administered via either the intraperitoneal (IP) (500 µL) or intranasal (IN) route (50 µL). Four groups of mice (n = 10 per group) were immunized on days 0, 7, and 21 with a multi-antigen formulation containing P5 (12.5 μg/mouse of each P5-12 and P5-302 variant) and P26 (25 μg/mouse).

Briefly, P5-12 and P5-302 correspond to representative alleles selected from the two major phylogenetic subfamilies of the P5 protein, chosen based on their frequency and diversity among clinical isolates. These alleles were identified through whole genome sequencing (WGS) of NTHi isolates and selected based on phylogenetic analysis of P5 amino acid sequences. P5 is a surface-exposed outer-membrane protein belonging to the OmpA family and plays a key role in host–pathogen interactions, notably through its ability to bind human complement regulatory protein factor H, thereby promoting bacterial survival by limiting complement-mediated lysis.

P26 is a highly conserved outer membrane protein with limited sequence variability across NTHi isolates and has been shown to induce both humoral and cellular immune responses. The P26-3 allele was similarly selected from WGS data based on its conservation and representation among clinical isolates. The combination of P5 and P26 was selected to target complementary mechanisms of protection, including inhibition of complement evasion and enhancement of antigen-specific immune responses, thereby increasing the breadth and effectiveness of the vaccine candidate. The sequences of P5-12, P5-302, and P26 have been previously described in our published work [15], and the corresponding nucleotide sequences are provided in the Supplementary Data. Briefly, coding sequences lacking the N-terminal signal peptide were amplified by PCR from NTHi isolates LNP32433 (P5-12), LNP31258 (P5-302), and LNP31429 (P26-3), respectively, and subcloned into the pET28b expression vector to generate recombinant proteins with a C-terminal hexahistidine tag. The resulting recombinant plasmids (pET-P5-12, pET-P5-302, and pET-P26-3) contained the corresponding genes in frame with the sequence encoding the C-terminal hexahistidine tag. Recombinant proteins were subsequently expressed in *E. coli* and purified by Ni-NTA affinity chromatography. Protein purity was assessed by SDS-PAGE analysis, as described in our published work [15].

For intranasal immunization, proteins were administered dropwise to lightly anesthetized mice to ensure delivery to the upper airways. Control mice received phosphate-buffered saline (PBS) alone.

For infection, mice were challenged intranasally on day 35 with 1 × 10^8^ CFU of NTHi isolate LNP31258 in 50 µL PBS under light anesthesia. Bacterial loads were quantified in bronchoalveolar lavage (BAL) fluid collected 6 h post-infection.

### 2.4. Responses in Mice to Vaccine Candidates

Bronchoalveolar lavage (BAL) was performed on mice by injecting and recovering 1 mL of physiological water. Cells from washes were first incubated with Fc block (anti-CD16/32) for 15 min on ice to prevent nonspecific antibody binding, followed by staining with fluorochrome-conjugated primary antibodies for 30 min in the dark at 4 °C. Fluorochrome-conjugated antibodies used included MHC-II (eFluor 450), CD45 (BV605), F4/80 (PE-Cy7), CD11b (Percp-Cy5.5), Ly6G (APC-Cy7), CD11c (APC) Ly6C (FITC), Siglec-F (PE-Texas Red) and CD3 (BV510). Antibodies specific to mice were purchased from BD Biosciences, Biolegend and eBioscience and were used at dilutions ranging from 1:100 to 1:200, according to the manufacturers’ recommendations.

Dead cells were excluded using a viability dye. Compensation controls and fluorescence minus one (FMO) controls were included to define gating strategies. Gating was performed sequentially on singlets, live CD45^+^ leukocytes, and specific myeloid subsets (macrophages: CD11b^+^F4/80^+^, neutrophils: Siglec-F^+^Ly6G^+^, monocytes: CD11b^+^Ly6C^+^, dendritic cells: CD11c^+^MHC-II^+^F4/80^+^; the gating strategy is provided in Supplementary Figure S1). In addition, total T cells were identified as CD45^+^CD3^+^ lymphocytes following exclusion of debris, doublets, and dead cells.

Data were acquired on LSRFortessa and analyzed using FlowJo software (version 10.10.0). Absolute cell numbers were calculated based on total BAL cell counts. Cell population frequencies and absolute numbers were compared between vaccinated and control groups using GraphPad Prism software, version 10. Data are presented as mean ± SEM (standard error of the mean).

Enzyme-linked immunosorbent assays (ELISAs) were also performed on dilutions of individual bronchoalveolar lavage samples from vaccinated and unvaccinated mice to detect IgG and IgA. BAL samples were analyzed in duplicate. Plates were blocked with 5% skim milk in PBS-Tween for 1 h at room temperature prior to incubation with samples. BAL samples were analyzed at a fixed dilution (1:500) to assess antigen-specific IgG and IgA responses. The ELISA plates were coated with 0.1 mL of 5 μg/mL of purified proteins P5-302, P5-12 or P26-3. ELISAs were performed using HRP-conjugated secondary antibodies (anti-mouse IgG and IgA; peroxidase-conjugated, affinity-purified; Abcam, Cambridge, UK) at a dilution of 1:5000, and signal detection was carried out using a chemiluminescent reaction, as previously described [19].

### 2.5. Statistical Analysis

The results are shown as the mean ± SEM. The statistical significance of differences between two samples was evaluated using Student’s *t*-test; *p* < 0.05 was considered statistically significant.

## 3. Results

### 3.1. Responses to Intraperitoneal Immunization in Mice

**Figure 1:**
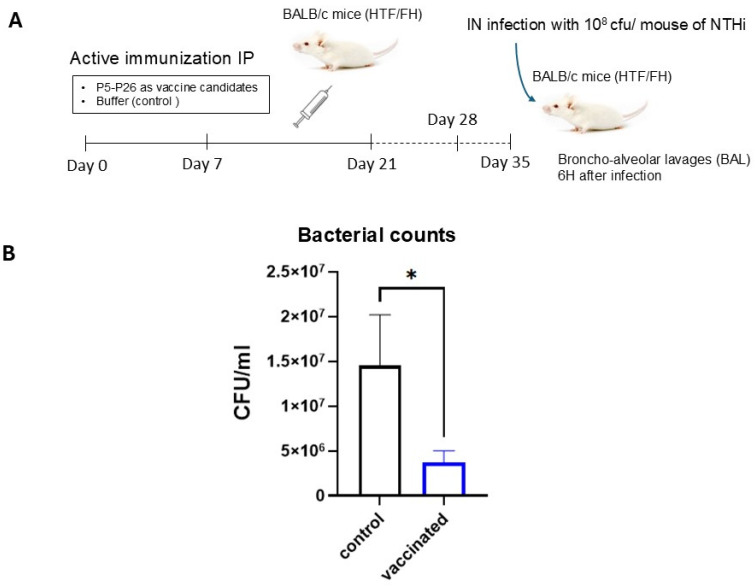
Protection induced by intraperitoneal immunization against intranasal NTHi challenge. (**A**) Schematic representation of the intraperitoneal immunization (IP) and intranasal (IN) challenge protocol. Mice were immunized on days 0, 7, and 21 with the P5–P26 multi-antigen formulation and challenged intranasally on day 35 with NTHi isolate LNP31258. (**B**) Bacterial burdens in bronchoalveolar lavage (BAL) fluids collected 6h after infection. Data are presented as mean ± SEM. Statistical significance was determined using Student’s *t*-test; * *p* < 0.05 was considered significant.

Mice immunized intraperitoneally with the multi-antigen formulation were challenged intranasally with NTHi LNP31258 isolate on day 35 post-immunization (Figure 1A). Six hours after infection, bacterial burdens in BAL fluids were significantly reduced in vaccinated mice compared with unimmunized controls (Figure 1B). Quantitative analysis showed a marked decrease in CFU counts in the vaccinated group, indicating enhanced early bacterial clearance.

Bacterial counts were measured in BAL fluids 6 h post-infection and were significantly lower in immunized mice compared with unimmunised controls (Figure 1B), suggesting a faster bacterial clearance.

**Figure 2:**
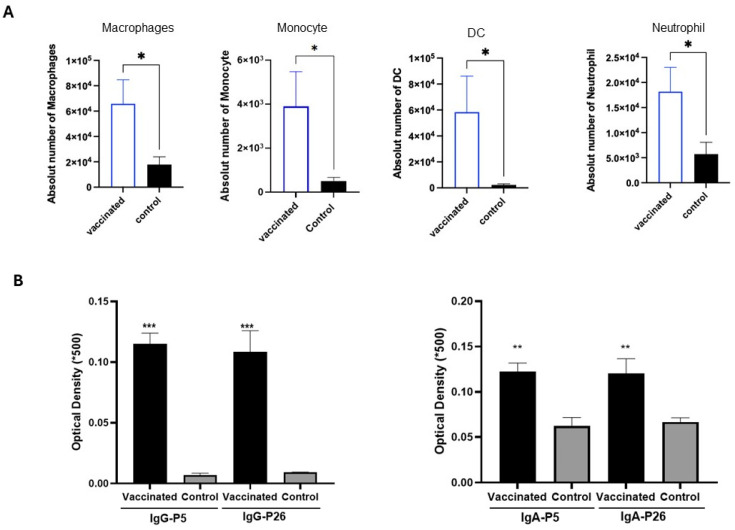
Immune responses induced by intraperitoneal immunization following intranasal NTHi challenge. (**A**) Recruitment of innate immune cells in BAL fluids 6 h after infection. Flow cytometry quantification shows increased numbers of macrophages, monocytes, dendritic cells (DCs), and neutrophils in vaccinated mice compared with controls. (**B**) Antigen-specific IgG and IgA levels in BAL fluids collected 24 h after infection. ELISAs show significantly higher antibody responses against P5 (alleles 302 and 12) and P26 proteins in vaccinated mice than in controls. Data are shown as mean ± SEM; statistical analysis was performed using Student’s *t*-test. The level of the significant difference between serogroups are indicated as follows (* *p* < 0.05, ** *p* < 0.01, *** *p* < 0.001).

Flow cytometric analysis of BAL samples showed that immunised mice exhibited significantly increased recruitment of innate immune cells 6 h post-infection (Figure 2A). These included macrophages, monocytes, dendritic cells (DCs) and neutrophils, indicating a rapid innate immune mobilization in the airways following challenge.

This enhanced cellular response suggests that immunization primes the airways for rapid innate immune activation upon NTHi exposure.

Finally, immunized mice displayed significantly higher levels of antigen-specific IgG and IgA in BAL 24 h post-infection compared with controls (Figure 2B). Antibodies targeted all purified antigens, P5-12, P5-302 (collectively referred to as P5), and P26-3, suggesting a strong local humoral response contributing to protection.

These findings indicate that intraperitoneal immunization induces both systemic priming and effective mucosal antibody responses upon respiratory challenge.

### 3.2. Responses to Intranasal Immunization in Mice

**Figure 3:**
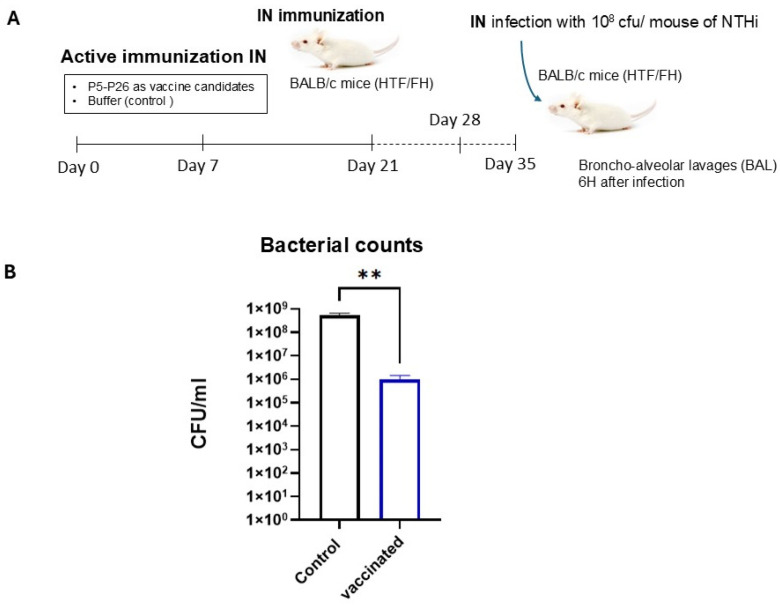
Protection induced by intranasal immunization against intranasal NTHi challenge. (**A**) Immunization and challenge timeline for intranasal vaccination. Mice received intranasal doses of the multi-antigen formulation on days 0, 7, and 21, followed by intranasal infection with NTHi LNP31258 on day 35. (**B**) Bacterial burdens in BAL fluids 6 h post-infection. Mice vaccinated intranasally exhibited significantly reduced bacterial loads compared with unvaccinated controls, indicating strong mucosal protection. Data are presented as mean ± SEM; The level of the significant difference between serogroups are indicated as follows (** *p* < 0.01).

Intranasally immunized mice were challenged intranasally on day 35 post-immunization (Figure 3A). As observed for intraperitoneal immunization, intranasally vaccinated mice exhibited significantly reduced bacterial loads in BAL 6 h post-infection compared with unimmunized controls (Figure 3B). The reduction in CFU counts confirms efficient early control of bacterial replication at the mucosal site.

**Figure 4:**
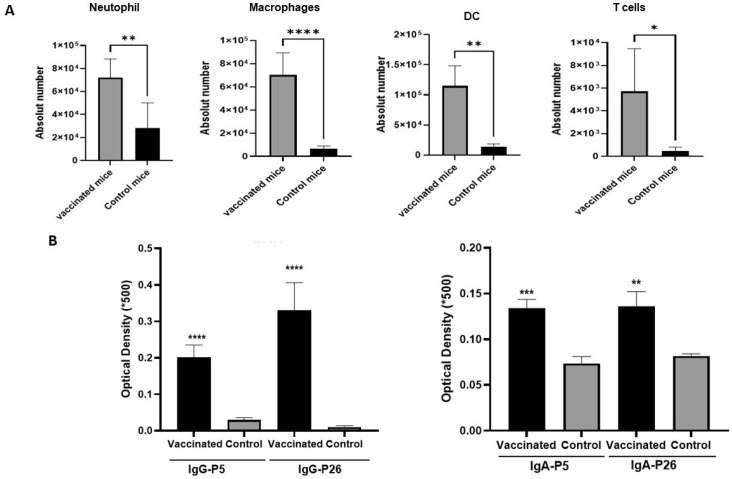
Immune responses induced by intranasal immunization following intranasal NTHi challenge. (**A**) Flow cytometry analysis of innate immune cell recruitment in BAL samples 6 h after infection. Intranasally vaccinated mice exhibited enhanced recruitment of macrophages, monocytes, DCs, and neutrophils compared with unvaccinated controls. (**B**) Antigen-specific IgG and IgA levels in BAL fluids 24 h after infection. ELISA results show robust mucosal antibody responses against P5 and P26 antigens in intranasally vaccinated mice. Data represent mean ± SEM. Statistical significance was assessed with Student’s *t*-test. The level of the significant difference between serogroups are indicated as follows (* *p* < 0.05, ** *p* < 0.01, *** *p* < 0.001, **** *p* < 0.0001).

Flow cytometry also revealed that intranasal immunization promoted enhanced recruitment of macrophages, monocytes, dendritic cells, and neutrophils to the airways following infection (Figure 4A). Notably, the magnitude of recruitment of neutrophils and monocytes appeared more pronounced following intranasal immunization compared with control mice, supporting enhanced local immune activation.

Immunization via the intranasal route elicited robust antigen-specific IgG and IgA responses in BAL (Figure 4B), consistent with induction of mucosal immunity. These antibody responses were significantly higher than in control mice and targeted all included antigens, reflecting successful induction of mucosal humoral immunity. Together, these data demonstrate that intranasal immunization efficiently promotes local antibody production and early innate immune recruitment, leading to improved bacterial clearance in the respiratory tract.

## 4. Discussion

The use of transgenic mice expressing human transferrin and factor H is critical to establish NTHi infection, as these bacteria rely on human sources for iron acquisition and on the binding of human factor H for complement evasion. This model therefore allows a relevant assessment of vaccine-induced protection.

Bacterial clearance was assessed at an early time point (6 h post-infection), corresponding to the peak of the inflammatory response in the airways. As NTHi infection is initiated at the level of the respiratory tract, early bacterial control is a critical determinant in preventing the progression from localized colonization to invasive disease. In this context, early protection represents a biologically relevant indicator of vaccine efficacy. Consistent with this, our previous work demonstrated protection at later time points in a systemic infection model, supporting the relevance of these findings for disease control beyond the initial stages of infection [6,15,20].

Both intraperitoneal and intranasal immunization routes significantly enhanced bacterial clearance and immune activation in the airways. A major strength of this vaccine strategy lies in the selection of conserved antigens that address the extensive heterogeneity of NTHi. P5 and P26 are widely distributed among clinical isolates and are functionally involved in host–pathogen interactions, including complement evasion and immune activation [6,15,20]. Induced antibodies likely contribute to bacterial clearance through opsonization, complement activation, and inhibition of bacterial adhesion to mucosal surfaces, key mechanisms for controlling NTHi colonization and infection [6].

Importantly, immunization promoted early recruitment of innate immune cells to the airways, a critical determinant of rapid bacterial clearance. Moreover, the recruitment of these antigen-presenting cells (APCs) likely contributes to the initiation and amplification of antigen-specific immune responses, including IgG and IgA production. The observed protection is likely associated with a combination of innate immune activation and antigen-specific antibody responses. In support of this, our previous work demonstrated that passive transfer of immune sera confers protection in a systemic infection model, highlighting a functional role for antibodies targeting P5 and P26 [6,15,20].

This coordinated innate response, together with antigen-specific antibodies, likely contributes to limiting bacterial replication and preventing the establishment and progression of infection.

Although no adjuvant was used in this study, the observed immune responses are unlikely to be driven solely by non-specific inflammatory effects. The synergistic effect previously observed following active immunization with the combination of P5 and P26, compared to each antigen alone, supports an antigen-specific mechanism. In addition, the protective effect demonstrated by the passive transfer of sera from immunized mice further highlights the role of antigen-specific antibodies in mediating protection [15].

Our results also highlight the importance of the innate immune response in early control of NTHi infection, with both intraperitoneal and intranasal immunization, indicating that the multi-antigen formulation can prime innate immunity regardless of the administration route.

Although both IgG and IgA responses were detected, the respective contribution of each isotype to protection could not be distinguished in this study; however, mucosal IgA is likely to play an important role following intranasal immunization. Given that NTHi infections initiate at mucosal sites, these findings support intranasal delivery as a highly relevant vaccination strategy. Together with our previous demonstration of systemic protection, these results highlight the versatility of the P5–P26 multi-antigen formulation and support its further development as a broadly protective NTHi vaccine candidate.

## 5. Conclusions

The multi-antigen P5-P26 vaccine induces robust mucosal and systemic immune responses and confers significant protection against pulmonary NTHi infection. These findings support further preclinical development of this vaccine strategy, with particular emphasis on intranasal delivery for the prevention of respiratory NTHi disease.

## 6. Patents

Patent application: EP 25305565.1. Multi-component vaccine candidates against non-typeable *Haemophilus influenzae.*

## Data Availability

The data supporting the findings of this study are available from the corresponding author upon reasonable request.

## Supplementary Materials

The following supporting information can be downloaded at https://www.mdpi.com/article/10.3390/vaccines14040357/s1, Figure S1: Gating strategies for flow cytometry on immune cells.
