# Supplementary material for: Multi-Antigen Protein Vaccine Confers Protection in a Murine Model Against Intranasal Haemophilus influenzae Challenge
Source: Vaccines (Basel). 2026 Apr 17;14(4):357. doi: 10.3390/vaccines14040357 (PMC13120526; doi:10.3390/vaccines14040357)
Supplement: Supplementary file 1 [file vaccines-14-00357-s001.zip › vaccines-4207003-supplementary.pdf]

The coding sequences used for recombinant protein expression were derived from selected NTHi isolates. Signal peptide-encoding regions were removed prior to cloning to allow expression of mature proteins. The resulting PCR-amplified fragments were subcloned into the pET28b expression vector to generate recombinant proteins with a C-terminal hexahistidine tag.

>P5-12 | isolate LNP32433

ATGAAAAAAACTGCAATCGCATTAGTAGTTGCTGGTTTAGCAGCAGCTTCA
GTAGCTCAAGCAGCTCCACAAGAAAACACTTTCTACGCTGGCGTTAAAGCTG
GTCAAGCATCTTTTCACGATGGACTTCGTGCTCTAGCTCGTGAACATAAAGTA
GGTTATCACCGTAATTCTTTCACTTATGGTGTATTCGGTGGTTATCAAATTTTA
AATCAAAATAACTTAGGTTTAGCGGTTGAATTAGGTTACGACGATTTCGGTCG
TGCCAAAGGTCGTGAAAAAGGTAGAACTGTTGCTAAACACACTAACCACGGT
ACTCACTTAAGCTTAAAAGGTAGCTATGAAGTGTTAGAAGGTTTAGATGTTTAT
GGTAAAGCAGGTGTTGCTTTAGTTCGTTCTGACTATAAATTCTACGATAATAAA
CGCATCGATAGTCACAGAGCACGTGCCTCTGGTTTATTTGCAGTAGGTGCAG
AATACGCAGTATTACCAGAATTAGCAGTTCGTTTAGAATACCAATGGCTAACT
CGCGTAGGTAAATACCGCCCTCAAGATAAACCAAATACCGCAATTAACTACAA
CCCTTGGATTGGTTCTATCAATGCGGGTATTTCTTACCGTTTTGGTCAAGGCG
AAGCGCCAGTTGTTGCAGCACCTGAAGTTGTAAGCAAAACTTTCAGCTTAAAC
TCTGATGTAACTTTCGCATTTGGTAAAGCAAACTTAAAACCTCAAGCACAAGC
AACATTAGACAGCATCTATGGCGAAATGTCACAAGTTAAAAGTGCAAAAGTAG
CTATTGCTGGTTACACTGACCGTATTGGTTCTGATGCGTTCAACGTAAAACTT
TCTCAAGAACGTGCAGATTCAGTAGCTAACTACTTTGTTGCTAAAGGTGTTGC
AGCAGACGCAATCTCTGCAACTGGCTACGGTAAAGCAAACCCAGTAACTGGT
GCAACTTGTGACCAAGTTAAAGGTCGTAAAGCGCTTATCGCTTGTCTTGCTCC
AGACCGTCGTGTAGAAATCGCGGTAAACGGTACTAAATAA

>P5-302 | isolate LNP31258

ATGAAAAAAACTGCAATCGCATTAGTAGTTGCTGGTTTAGCAGCGGCTTCA
GTAGCTCAAGCAGCTCCACAAGAAAACACTTTCTACGCTGGCGTTAAAGCTG
GTCAAGGATCTTTCCATGATGGTATTAACAATAATGGCGCAATTAAAGGGGAT
TTAACACTTGGTTATGGTTACAGACGCAATACTTTCACTTATGGTGTATTTGGT
GGTTACCAAATTTTAAATCAAGATAATTTTGGTTTAGCTGCTGAATTAGGTTAC
GACAATTTTGGTCGTGTAAAATTTCGTGCAGAAGGAAAAACTAAAGCTAAACA
TACTAACCACGGTGCGCACTTAAGCTTAAAAGGCAGCTATGAAGTGTTAGAC
GGTTTAGATGTTTATGGCAAAGCAGGTGTTGCTTTAGTACGTTCTGATTATAA
ATTTTATGAAGATGCAAACGGTACTCGTGACCACAAGAAAGGTCGTCACACA
GCACGTGCCTCTGGTTTATTTGCAGTAGGTGCAGAATACGCAGTATTACCAG
AATTAGCAGTTCGTTTAGAATACCAATGGCTAACTCGCGTAGGTAAATACCGC
CCTCAAGCTAAACCAAATACCGCAATTAACTACAACCCTTGGATTGGTTCTAT
CAATGCGGGTATTTCTTACCGTTTTGGTCAAGGCGCAGCACCAGTTGTAGCA
GCACCTGAAGTTGTAAGCAAAACTTTCAGCTTAAACTCTGATGTAACTTTCGC
ATTTGGTAAAGCGAACTTAAAACCTCAAGCACAAGCAACATTAGACAGCATCT
ATGGCGAAATGTCACAAGTTAAAAGTGCAAAAGTAGCTGTTGCTGGTTACACT
GACCGTATTGGTTCTGATGCGTTCAACGTAAAACTTTCTCAAGAACGTGCAGA
TTCAGTAGCTAACTACTTTGTTGCTAAAGGTGTTGCAGCAGACGCAATCTCTG
CAACTGGCTACGGTAAAGCAAACCCAGTAACTGGCGCAACTTGTGACCAAGT
TAAAGGTCGTAAAGCACTTATCGCTTGTCTTGCTCCAGACCGTCGTGTAGAAA
TCGCGGTAAACGGTACTAAATAA

>P26-3 | isolate LNP31429

ATGAAAAACATCGCAAAAGTAACCGCACTTGCTTTAGGTATTGCACTTGC
TTCAGGCTATGCTTCCGCTGAAGAAAAAATTGCTTTCATTAATGCAGGTTA
TATTTTTCAACATCACCCAGATCGCCAAGCAGTAGCAGATAAACTTGATGC
TGAATTTAAACCTGTAGCTGAGAAATTAGCAGCAAGCAAAAAAGAAGTTGA
TGATAAAATTGCTGCTGCTCGTAAAAAAGTAGAAGCAAAAGTTGCGGCTTT
AGAAAAAGATGCACCTCGCTTACGTCAAGCTGATATTCAAAAACGCCAACA
GGAAATTAATAAATTAGGTGCGGCTGAAGATGCTGAATTACAAAAACTAAT
GCAAGAACAAGATAAAAAAGTTCAAGAATTCCAAGCTCAAAATGAAAAACG
TCAAGCTGAAGAACGTGGTAAATTATTAGATAGCATTCAAACTGCGACAAA
TAATTTAGCAAAAGCAAAAGGTTATACTTATGTGCTTGATGCAAATTCAGTT
GTATTTGCGGTAGAGGGTAAAGATATTACTGAAGAAGTATTAAAATCTATC
CCTGCTTCTGAAAAAGCACAAGAGAAAAAATAA
