# Supplementary figures and images for: Multi-Antigen Protein Vaccine Confers Protection in a Murine Model Against Intranasal Haemophilus influenzae Challenge
Source: Vaccines (Basel). 2026 Apr 17;14(4):357. doi: 10.3390/vaccines14040357 (PMC13120526; doi:10.3390/vaccines14040357)

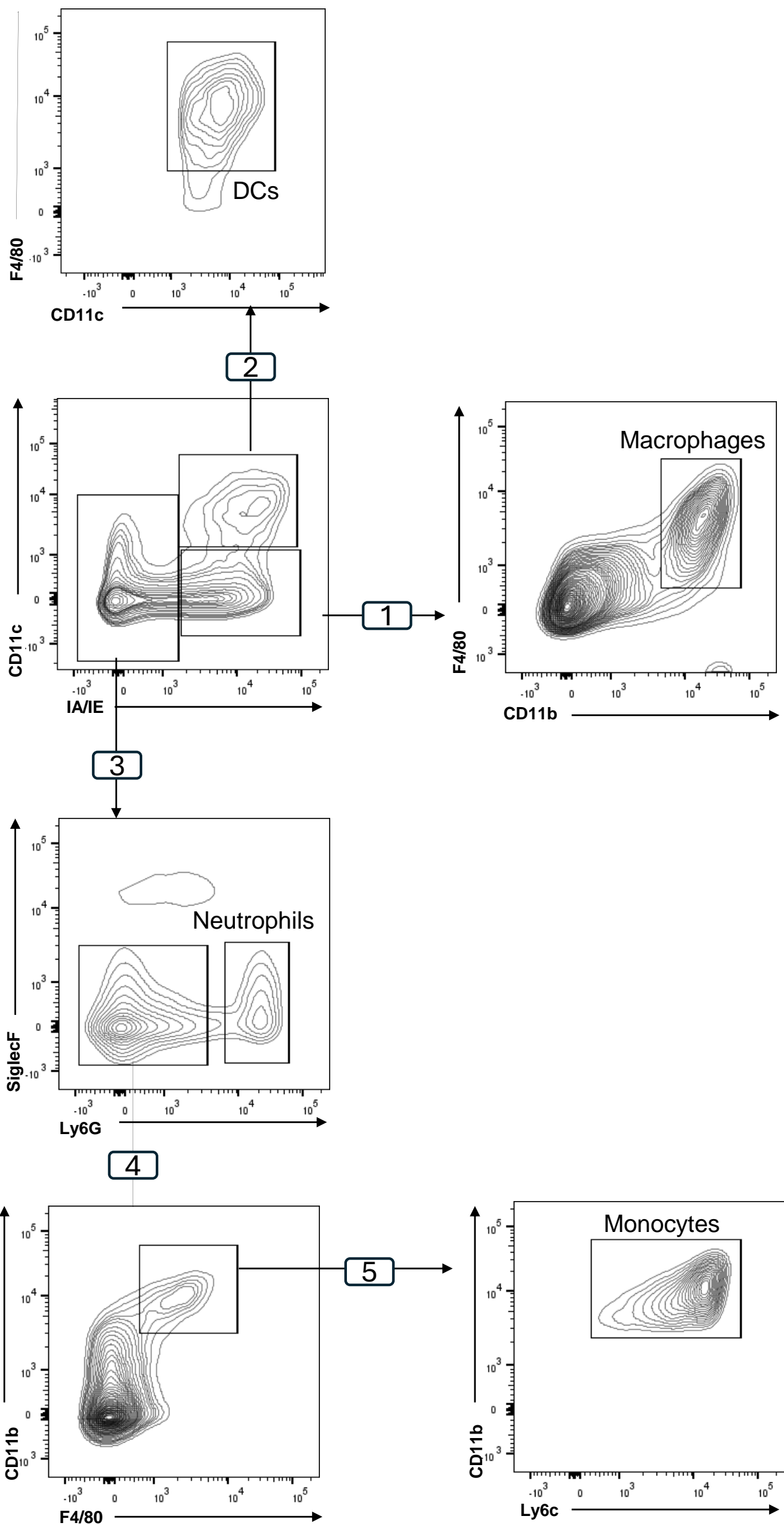

**Supplementary figure S1:** Gating strategies for flow cytometry on immune cells

Supplement: Supplementary file 1 [file vaccines-14-00357-s001.zip › vaccines-4207003-Figure S1.pdf]
